# Flow of long chain hydrocarbons through carbon nanotubes (CNTs)

**DOI:** 10.1038/s41598-021-90213-7

**Published:** 2021-05-26

**Authors:** Pranay Asai, Palash Panja, Raul Velasco, Milind Deo

**Affiliations:** Department of Chemical Engineering, University of Utah, Salt Lake City, USA

**Keywords:** Energy science and technology, Engineering, Nanoscience and technology

## Abstract

The pressure-driven flow of long-chain hydrocarbons in nanosized pores is important in energy, environmental, biological, and pharmaceutical applications. This paper examines the flow of hexane, heptane, and decane in carbon nanotubes (CNTs) of pore diameters 1–8 nm using molecular dynamic simulations. Enhancement of water flow in CNTs in comparison to rates predicted by continuum models has been well established in the literature. Our work was intended to observe if molecular dynamic simulations of hydrocarbon flow in CNTs produced similar enhancements. We used the OPLS-AA force field to simulate the hydrocarbons and the CNTs. Our simulations predicted the bulk densities of the hydrocarbons to be within 3% of the literature values. Molecular sizes and shapes of the hydrocarbon molecules compared to the pore size create interesting density patterns for smaller sized CNTs. We observed moderate flow enhancements for all the hydrocarbons (1–100) flowing through small-sized CNTs. For very small CNTs the larger hydrocarbons were forced to flow in a cork-screw fashion. As a result of this flow orientation, the larger molecules flowed as effectively (similar enhancements) as the smaller hydrocarbons.

## Introduction

Shale and tight formations have been developed rapidly in recent years for the economic exploitation of hydrocarbon fluids despite the lack of adequate understanding of fluid flow in these porous media^1–6^. These reservoirs are characterized by extremely low permeabilities and small pore sizes in the order of nanometers. It is recognized that recoveries are low. Addressing the knowledge gap of hydrocarbons flow in nanosized pores will help resolve the significant challenges of improving recoveries.

It is well established in the literature that the properties of fluids (thermodynamic and flow) under confinement deviate significantly from their bulk properties, depending on the extent of confinement^7,8^. For the flow under confinement, Darcy’s law^9^, which defines a relationship between flow rate and pressure in porous media, may not be applicable^10^. Studies on water flow dominate the experimental realm of flow in confinement. Majumder et al.^11,12^ reported flow rates of water in 7 nm diameter CNTs to be four to five orders of magnitude higher than expected continuum flows. Holt et al.^13^ found the flow to be three orders of magnitude faster than values calculated using continuum hydrodynamics models in 2 nm diameter CNTs. Whitby et al.^10,14^ also found enhanced flow in CNTs with water, and they reported the same trend for Ethanol and Decane. Verweij^15^ built on Holt’s group^13^ findings and concluded that the fast flow in CNT is due to the smooth surface of CNT, which led to ultralow friction with the fluid molecules. Mattia et al.^16^ performed experiments demonstrating enhanced permeability of water in CNT.

Though experimental results provide a better overview of this concept, setting-up experiments and fluid flow measurement at a nano-scale face many challenges. Therefore, flow modeling using molecular simulation plays an important role in developing a preliminary understanding of the molecular motion and eventually leads to development of better experimental systems. Some of the applications of this modeling include the design of nanotube-based biosensors^17^, nanomembranes^18,19^, gas storage, and separation^20–23^. Molecular dynamic (MD) simulation using appropriate force fields is an efficient tool to calculate equilibrium properties like density in confinement, self-diffusion coefficient, and molecular orientation. The results from MD simulations are only as good as the chosen force-field. Each force-field has its advantages and disadvantages, for some are developed to predict structures, whereas some are good at predicting specific thermodynamic properties. A universal force field to predict all properties of interest is not available, and often, custom force fields are necessary. Hence, it is crucial to carefully choose an appropriate force field, depending on the scope of the study. Also, it should be noted that most of the properties, like the self-diffusion coefficient, are highly sensitive to temperature^24,25^.

On the simulation part of non-equilibrated systems, most experimental results for flow enhancement of water inside CNT have been successfully replicated using MD modeling. Joseph et al.^26^ concluded that the fluid wall interaction causes water enhancement in CNT. Thomas et al.^27,28^ and Velasco et al.^29^ showed that the enhancement of flow depends on the size of the CNT and diminishes as the diameter is increased. Alexiadis et al.^30^ concluded that all the fluid properties vary with the CNT diameter change irrespective of surface functionality. Ebrahimi et al.^31^ showed nonuniform CNT’s effect on water flow by creating junctions in the tube. All these studies reached a consensus conclusion of enhanced flow of water in CNT, which agrees with the experimental results. This enhancement has been ascribed to frictionless flow and slip. However, there are limited studies on the flow of long-chain hydrocarbons in CNT. It is important to understand how complex hydrocarbon molecules behave and flow under confinement. It is hypothesized that for a complex molecule, there would be multiple effects (confinement, molecular geometry, and pore geometry) attributing to its behavior in confinement. Santiso et al.^32^ discussed the geometric constraints on long-chain hydrocarbons under CNT confinement and reported hindering rotational isomerism of hydrocarbons in tight spaces. In this study, the effect of confinement on the flow of various long-chain hydrocarbons in nanosized CNT is examined to augment the understanding of flow behavior under non-equilibrated conditions. The system consists of a simple cylindrical pore model made of CNT and flow is simulated using a molecular dynamic simulator LAMMPS^33^. OVITO (version 3.3.0)^34^ was used to create all the molecular figures for this study.

## Results

### Bulk properties

The OPLS-AA force-field for hydrocarbon gives close enough density values for the corresponding bulk hydrocarbons (see Table 1) with decane deviating the most but still in reasonable range of less than 6% error. These calculations are described in more detail in the “Methods” section and in the Supplementary Information. The OPLS-AA force field does not accurately predict the self-diffusion coefficient for the hydrocarbons when compared to the experimental value. Still, the values obtained from MD simulation are in agreement with previously reported values in the existing literature. This shows that the force-fields are customized to predict only the particular properties accurately. We chose this specific force-field because of its ability to predict densities and diffusivities reasonably well.

**Table 1:** Comparison of simulation values to existing literature values.

	Bulk density (kg/m^3^)			Diffusivity (m^2^/s)		
	Experimental	MD	Deviation %	Previous MD	MD	Deviation %
Hexane	655.8^23^	636.45	2.95	3.54E−09^35^	2.82E−09	5.68
Heptane	679.5^36^	659.33	2.97	–	2.22E−09	–
Decane	725.5^37^	702.98	3.10	–	9.35E−10	–

### Inside the CNT

#### Equilibrated simulations

**Figure 1:**
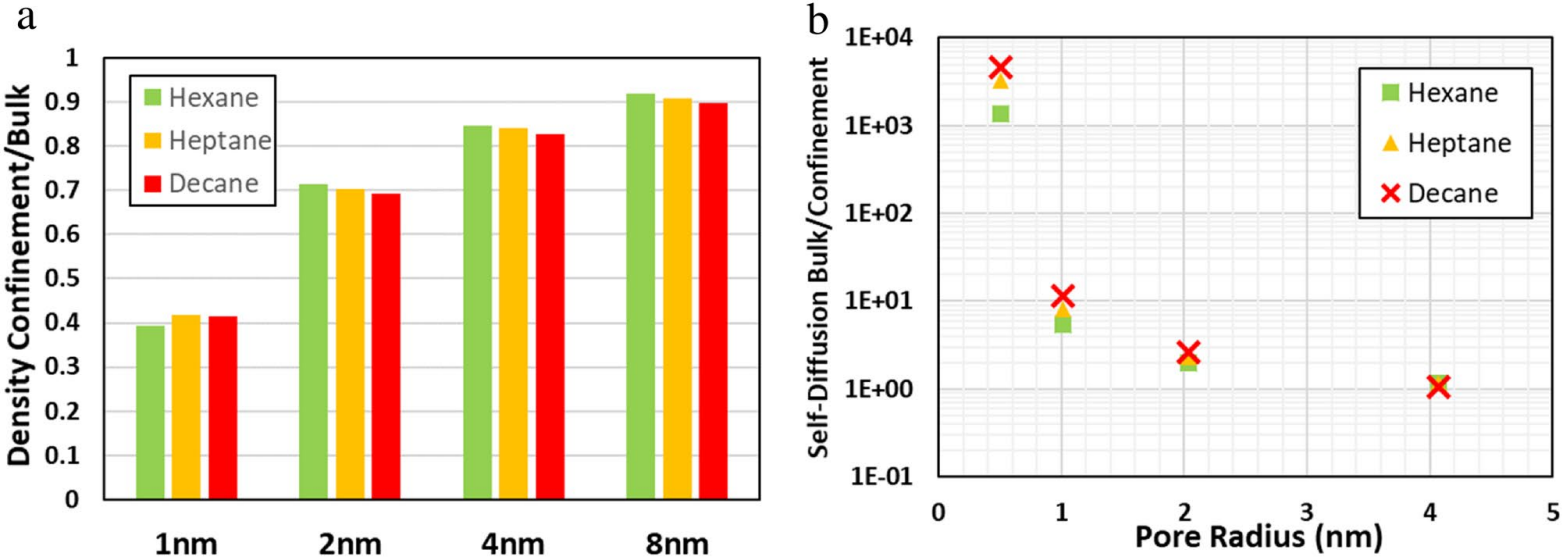
Effect of confinement on the fluid inside the CNT, (**a**) ratio of the density of fluid under over density of the fluid in bulk, calculated using MD simulation at 1 atm, (**b**) ratio of self-diffusion coefficient in bulk over confinement obtained from MD simulation. Created using OVITO (ver:3.3.0).

It was observed that the density inside the CNT varies significantly with the size of the CNT. The smallest CNT had the maximum reduction in density, and as the size of the CNT is increased, the density approached the bulk density of the fluid (see Fig. 1a). The reduction in density is postulated because of the excluded volume and the curvature of CNTs^27^. The excluded volume is defined as the region around a given atom where no other atom can enter. This parameter becomes quite dominant when the CNT size is comparable to the size of the molecule. However, as the size of the CNT increases, the excluded volume effect diminishes, and the densities tend to get closer to the bulk densities. In this study, density variations due to geometric constraints on the long-chain hydrocarbons are also observed. The sheer curvature of the CNT wall at a smaller diameter also affects the excluded volume allowing less space for the fluid molecules. It forces them to align in a particular direction. Due to geometric constraints, the long-chain hydrocarbons preferably align themselves along the length of CNT when the diameter of the CNT is smaller than the length of the hydrocarbons. At any given cross-section in 1 nm CNT, only one molecule of hydrocarbon is present.

Figure 1b shows the ratio of experimental bulk self-diffusion and confined (pore) self-diffusion. The higher ratio corresponds to the reduction in diffusivity in confinement. The diffusivity in confinement also varies considerably due to the effects similar to those observed for the density change. The geometric effects are vividly apparent for the hydrocarbons, with self-diffusivity decreasing by several order of magnitudes as compared to bulk. As the hydrocarbons are coiled up in the smallest CNT, and each molecule has two immediate neighbors, the self-diffusion decreases drastically with decane having the least diffusivity^38^. As the CNT diameter is increased, the ratio decreases, and the diffusivity value approaches the bulk.

**Figure 2:**
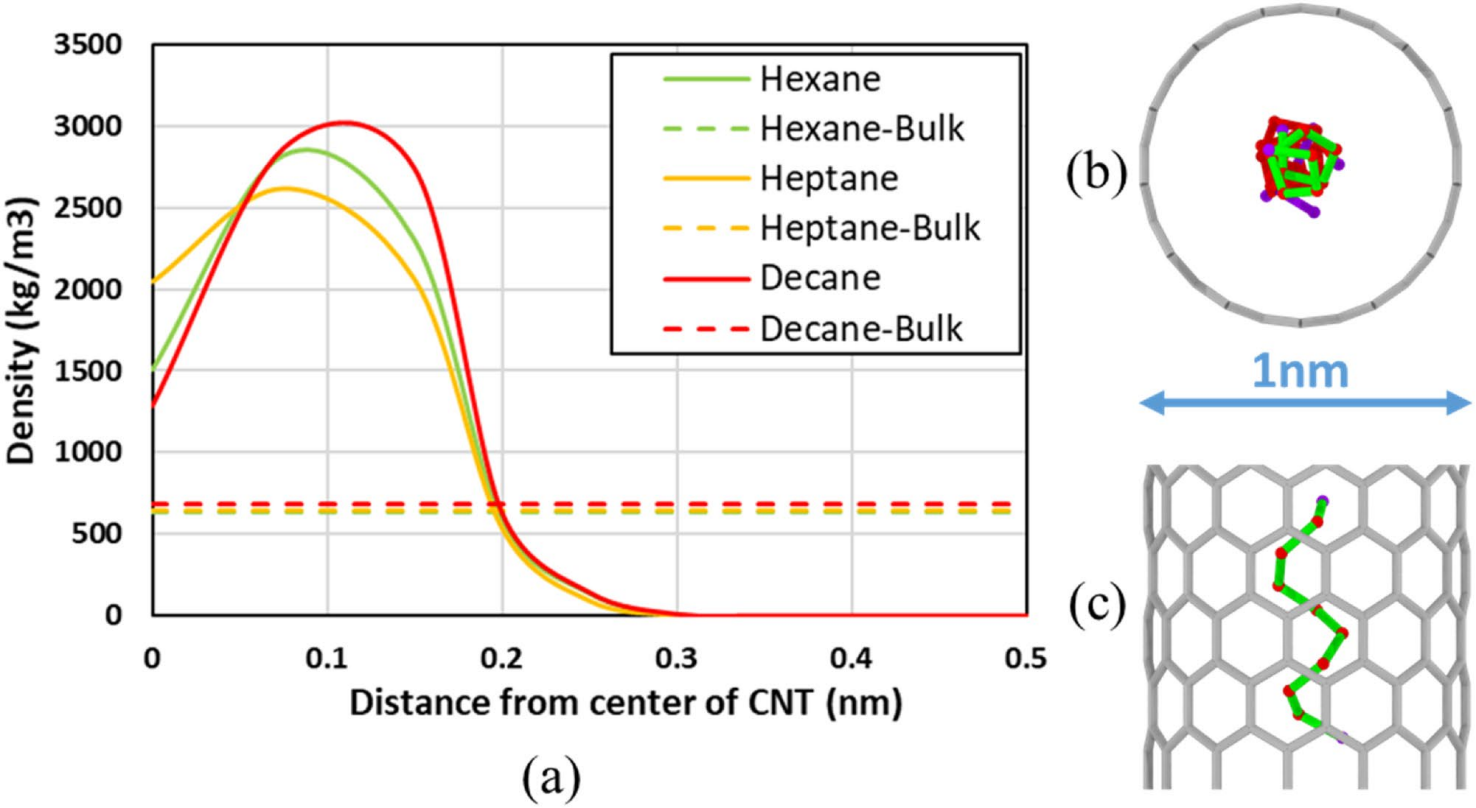
(**a**) Effect of confinement on the cross-sectional local density of fluids under confinement compared to the bulk density for 1 nm diameter CNT at 1 atm. (**b**) Cross-section of decane (carbon chain only) in 1 nm CNT, and (**c**) side view of decane (carbon chain only) in 1 nm CNT. Red represents carbon, and Purple represents terminal carbon for the given Decane molecule. The green color represents the highlighted molecule from two different perspectives. Created using OVITO (ver:3.3.0).

Apart from the change in overall density under confinement, radial variations are also observed. The cross-sectional local density profile shows some exciting results with the prominent effect of excluded volume, curvature of CNT, and the geometric effect of fluid molecules in the 1 nm diameter CNT (see Fig. 2a). Here, each hydrocarbon’s length is comparable to the diameter of the tube leading to geometric constraints. This causes the carbon backbone in long-chain hydrocarbons to coil up into spirals inside the CNT (Fig. 2b,c). The hydrogens in the hydrocarbon point inwards and outwards, causing low-density regions at the center and near the walls. Due to this confinement effect (excluded volume and geometric effect), the densities are much higher near the center when compared to bulk. In Fig. 1, decane molecule is highlighted (shown in green), and two different perspectives are offered to visualize the long-chain hydrocarbon coiling. Depending on how the molecules are coiled, the radial density for the different hydrocarbons changes.

**Figure 3:**
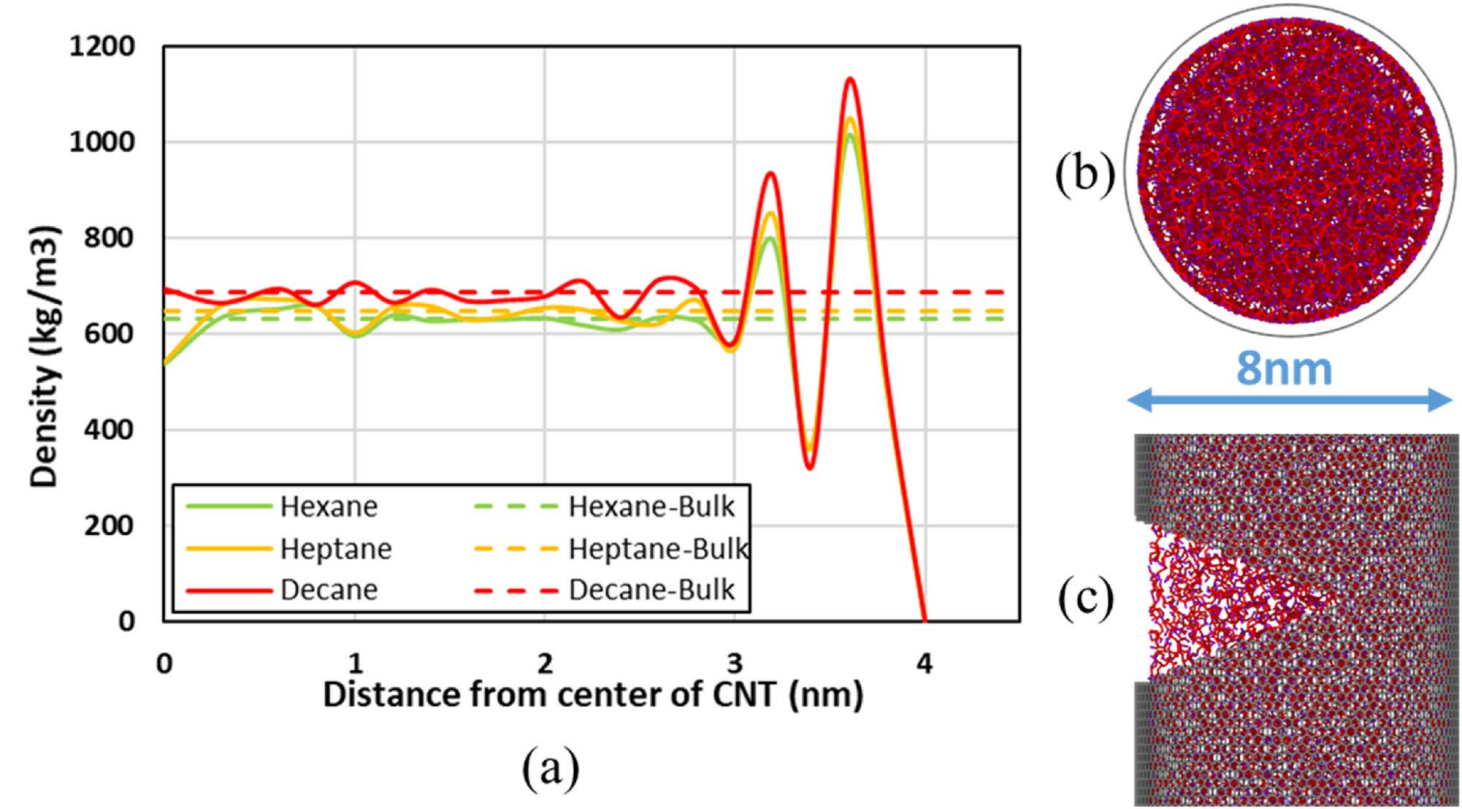
(**a**) Effect of confinement on the cross-sectional local density of fluids under confinement compared to the bulk density for 8 nm diameter CNT at 1 atm, (**b**) cross-section of decane (carbon chain only) in 8 nm CNT, and (**c**) side view of decane (carbon chain only) in 8 nm CNT. Red represents carbon, and Purple represents terminal carbon for the given Decane molecule. A small slit is cut into the CNT to observe the arrangement of hydrocarbon inside. Created using OVITO (ver:3.3.0).

In the case of 8 nm CNT, as the tube is much bigger than the molecular size, all the effects near the center are diminished, and the fluid retains the property of the bulk. However, a significant variation of the density is observed near the wall. A dense ring of hydrocarbon is formed near the wall, which is caused due to the adsorption at the wall (see Fig. 3a–c). As we move toward the center of the tube, the effect of the wall diminishes. Thus, the densities tend towards the bulk density of the fluid. The more significant variations for the hydrocarbons can be attributed to the carbon chain’s length, with decane (longest chain) having the maximum effect.

#### NEMD simulation

NEMD (Non-equilibrated Molecular Dynamics) simulations were performed to assess the flow performance of these various fluids under confinement. For a given size, the tube was split into three regions, “Force Region,” “Buffer Region,” and “Calculation Region.” A constant force was applied on each carbon atom present in the force region (and on the oxygen atom for water simulations) to simulate a pressure drop of one atmosphere per Angstrom of the entire tube length. Flow rates of the molecules were calculated (in the calculation region) after fully developed flow (constant velocity) was achieved. Since all the fluids have different viscosities, to make the results comparable, an equivalent flowrate was plotted for the different radii of CNTs. The equivalent flowrate is defined as the product of flow rate and viscosity (calculations shown in “Simulation procedure” section). In this study, we used constant viscosity (bulk viscosity) for all the cases. This assumption was necessary to get an estimate on the trend of flow enhancement for the different fluids. Determining viscosities under confinement was not one of the study objectives and was beyond the scope of this paper.

**Figure 4:**
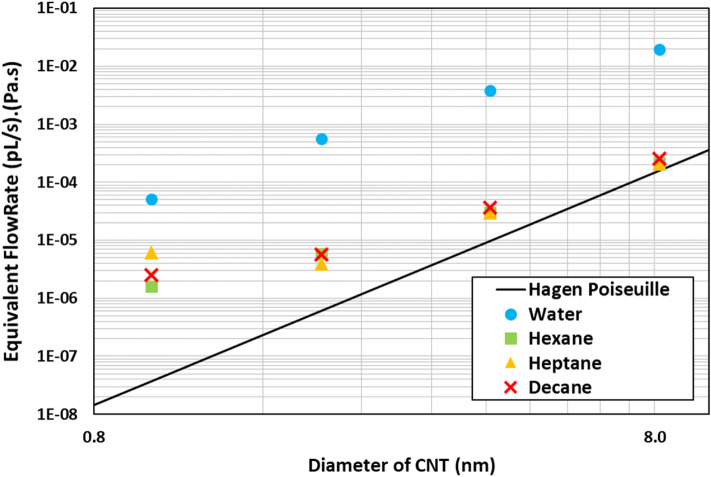
Flowrate of different fluid plotted against the radius of the CNT on a Log–Log scale. The black line represents the continuum line derived from Haggen–Poiseuille flow. The solid points represent the work produced from this study. The dashed lines are backward extrapolation to predict the flow rate at lower radii.

Figure 4 shows the equivalent flowrates of various fluids for the different diameters of the CNTs. The solid black line represents the equivalent flowrate results obtained from the Hagen–Poiseuille equation when plotted against the radius of the tube at a given pressure of 1 atm/A. The various points (square, triangle, circle, and cross) represent the equivalent flowrates obtained from the MD simulations. For comparative reasons, results for water are also plotted. It was observed that water had a significant enhancement when the pore size was 1 nm in diameter, and it starts to diminish as pore size is increased. The results for water in this study are in line with the experimental results presented by Holt et al.^13^ and the simulation results presented by Thomas et al.^27^ as shown in Table 2. Holt et al. reported the flow to be three orders of magnitude enhanced for 2 nm diameter CNTs. Thomas et al. reported about enhancements of about 500 for pore sizes between 1.6 and 5 nm diameter and enhancements of about 100 for 5–10 nm diameter tubes for water flow in CNT using reflective particle membrane method to simulate flow.

**Table 2:** Comparison for flow enhancement of water in CNT.

This study (simulation)		Holt et al.^13^ (experiment)		Thomas et al.^27^ (simulation)	
Diameter (nm)	Enhancement	Diameter (nm)	Enhancement	Diameter (nm)	Enhancement
1	1370	–	–	1.6–5	~ 500
2	925	2	~ 1000		
4	390	–	–		
8	125	–	–	5–10	~ 100
–	–	–	–	> 10	1–10

**Figure 5:**
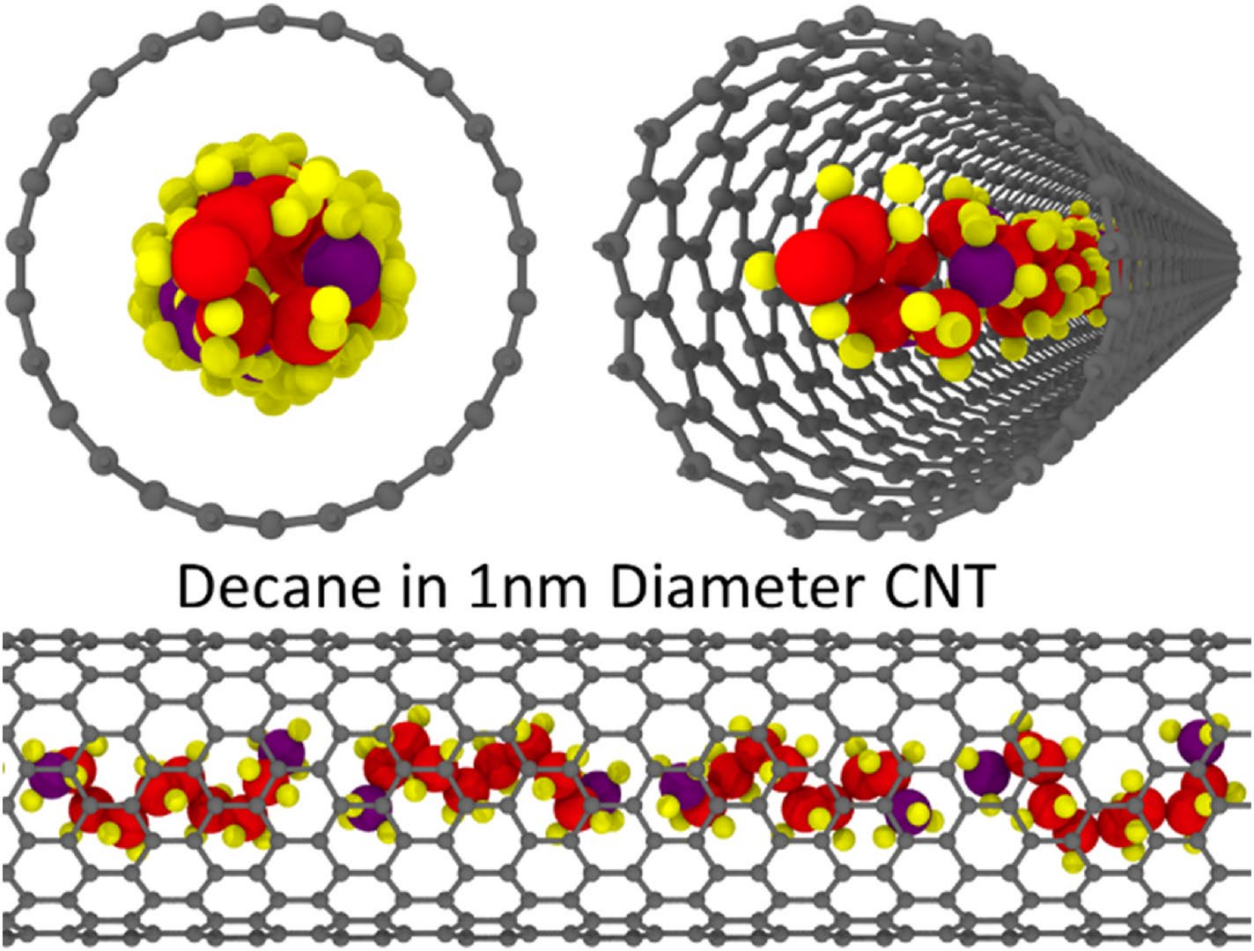
Different viewing angles for the flow of decane in 1 nm diameter CNT. Created using OVITO (ver:3.3.0).

**Figure 6:**
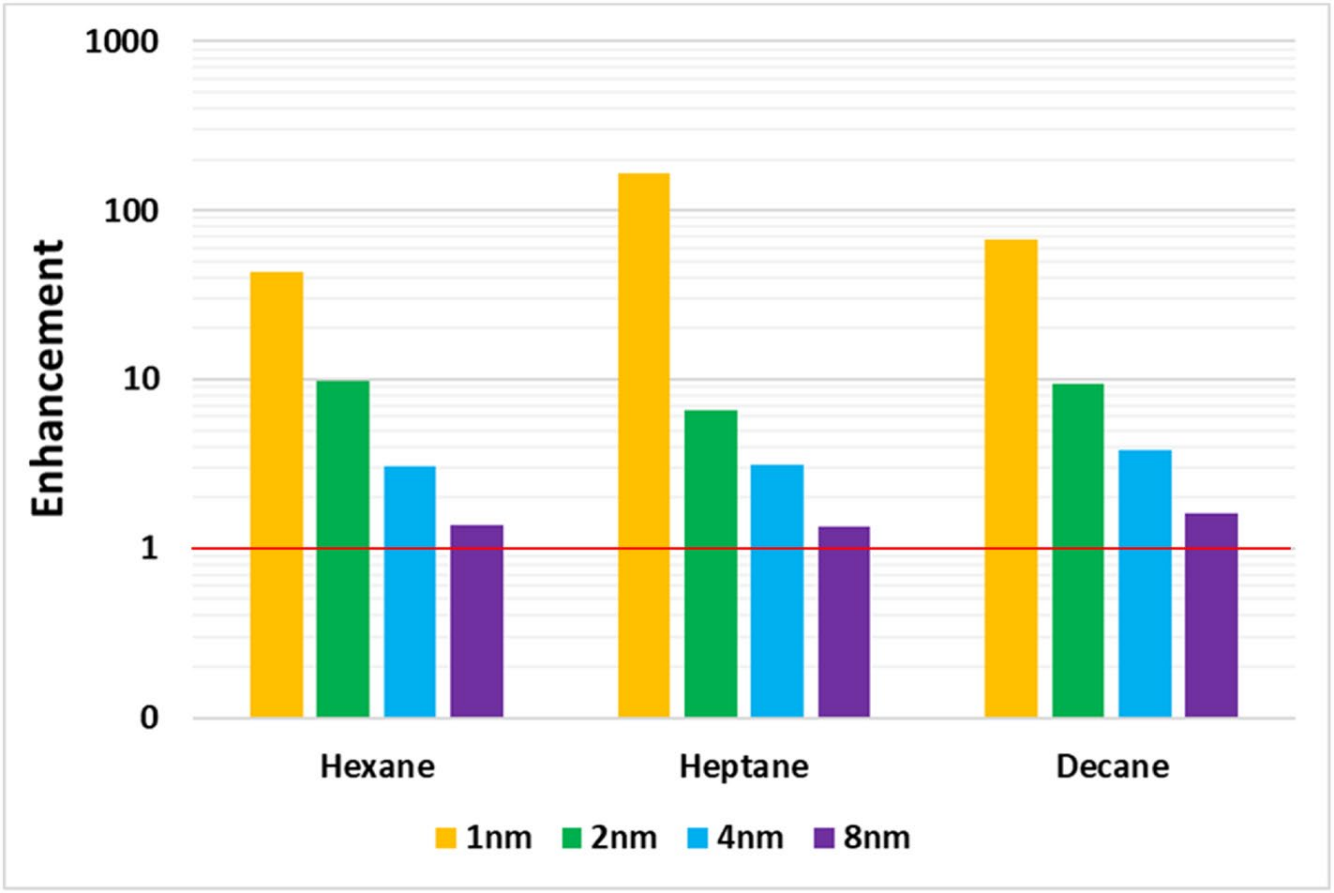
Enhancement factor for the flow of hydrocarbons in different sizes of CNT. The redline indicates no enhancement (i.e. following Hagen Poiseuille equation).

The flowrates for hydrocarbons are enhanced at smaller radii, but as the radii of the CNT increases, the enhancement starts to diminish significantly. This further validated that the hydrocarbon flowrates are not as enhanced on hydrophobic surfaces^14,38^ compared to water. However, in smaller CNTs, what we see in our simulations is the interaction of the molecular geometry with the pore’s geometry. The hydrocarbon molecules inside 1 nm diameter CNT form a single file and flow in a cork-screw fashion (see the example of Decane in Fig. 5). This is due to the geometric effect of the fluid molecules and the tube, which streamlines the flow in the pore and enhances it. Falk et al.^39^ also observed molecular reorientations as the pore sizes decreased. As the CNT pore radius is increased, the long-chain molecules no longer have a restriction for the orientation, and hence the flow enhancement diminishes (see Fig. 6). Only moderate enhancements are observed for all hydrocarbons (maximum being 100). It is also observed that heptane shows the highest enhancement among these sets of compounds in the 1 nm diameter CNT. This may be because of the non-symmetric structure of heptane (odd number of carbon atoms). However, as the CNT diameter is increased, this non-symmetric molecule effect fades away. Further investigation is needed to ascertain this effect. It should be noted that the results are affected by the force fields and the methods used to implement the simulations. Hence, caution should be used in interpreting the results of these simulations broadly.

Figure 6 shows that as the radius of the tube increases, the effect of confinement would decrease and eventually match the continuum line. The results show that under nano-confinement several effects, including excluded volume of molecules, geometric constraints of wall and fluid, and the fluid-wall interactions affect transport properties.

## Summary

We have presented a study of the changes in properties of long-chain hydrocarbons (hexane, heptane and decane) in CNTs. The densities of hydrocarbons in CNTs decrease as the size of the tube decreases because of excluded volume. Self-diffusion coefficients are a few orders of magnitude lower under confinement in comparison to the bulk. Values of these properties approach bulk values as the CNT diameters increase. NEMD simulations of pressure-driven flow show moderate enhancements in comparison to Hagen-Poiseuille flow (maximum of about 100 for 1 nm diameter) for all of the hydrocarbons. Flow approaches continuum as the diameter of CNT approaches 10 nm. Enhancements are much lower in comparison to water flow in CNTs. Interesting features related to orientation of hydrocarbons inside the tight geometrical confines of the CNTs are observed. It should be noted that results of the molecular dynamic simulations are affected by the force fields employed and the manner in which the methods are implemented.

## Methods

### Simulation procedure

Before the simulations are carried out, it is vital to validate the accuracy of the force fields. To do this, bulk simulations were carried out, and once they were deemed accurate, the flow simulations were performed. The information about the force-field is provided in the Supplementary Information.

### Bulk simulation

Initial simulations were carried out to simulate the bulk environment and determine the bulk properties for the simulated fluids. This was achieved by simulating a cubic simulation box with the side of five nanometers and with a three-dimensional periodic boundary. It was filled with the number of fluid molecules ($$n$$) corresponding to the bulk density ($$\rho )$$ of the fluid for the given volume ($$V)$$ calculated using (Eq. (1)), where “*M*” is the molecular weight of the fluid molecule and “*N*_*A*_” represents the Avogadro’s number. All the initial positions for the molecules were built using Moltemplate.

1 $$n = \frac{{V\rho N_{A} }}{M}.$$

This system was simulated using LAMMPS under alternating NPT and NVT conditions to obtain the bulk density and other bulk properties for the given fluid simulated using different force fields. The system was operated at 300 K, and each thermostat was simulated for one nanosecond each, starting with NPT following by NVT and repeated for three cycles^40^. All the density and diffusion coefficient measurements were made at the end of the last cycle under NVT conditions. These results were compared with the results from the existing literature.

The timestep for all the measurements was kept constant at 1 ps (picosecond). For the calculation of density, averaged measurements were done over every 50,000 timesteps (0.05 ns).

The diffusivities were calculated by obtaining the slope of the mean squared displacement of the molecules obtained over a given time as per Eq. (2)^41^, where, “*d*”, is the dimensionality (three for 3D) and the limit calculates the slope of average MSD of all the molecules over very long period of time, to ensure accuracy of results.

2 $$D = \frac{1}{2d}\mathop {\lim }\limits_{t \to \infty } \frac{{r\left( {t_{o} + t} \right) - r\left( {t_{o} } \right)}}{t}.$$

### Tube simulation

**Figure 7:**
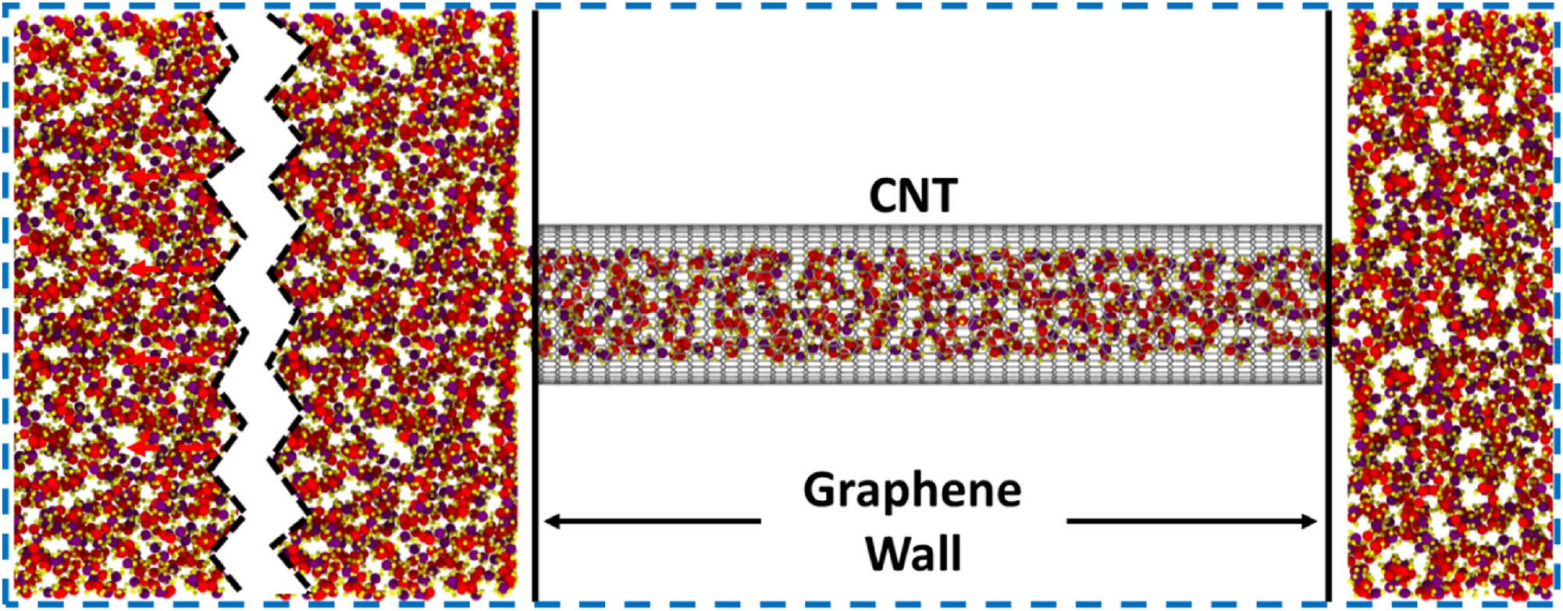
Setup to fill the CNT with Hexane by attaching it to a large reservoir and allow it to equilibrate. The Zig-zag line represents the kink to show the big reservoir. The blue dashed line represents the periodic boundary. Created using OVITO (ver:3.3.0).

As it is speculated that density under nano-confinement varies^42^, it is important to accurately measure the density for each fluid confined in all the CNTs. The length over diameter ratio (L/D) of 7 was maintained for all sizes to avoid entrance effects. To prepare the CNT for flow simulations, a large reservoir (100 times the volume of the CNT) of the fluid was initially attached to the CNT (Fig. 7) along with two graphene sheets at the end of the CNT, which act as walls. A hole was cut into both the walls to enable the fluid to enter the CNT. The system was allowed to be equilibrated for more than 20 ns at 300 K under the NVT thermostat^43,44^. This system had a periodic boundary condition (represented as a blue dotted line in Fig. 7) in all the directions to allow the CNT to be filled faster. When the number of fluid molecules in the CNT became constant, the density $\left( {\rho_{T} } \right)$ inside the CNT was obtained by counting the number of molecules $\left( {n_{T} } \right)$ and the volume of the CNT (calculated from its length and radius) as per Eq. (3), where, “*A*_*T*_” and “*L*_*T*_” represents the cross-sectional area and length of the CNT.

3 $$\rho_{T} = \frac{{n_{T} M}}{{A_{T} L_{T} N_{A} }}.$$

While the simulations were running, it was ensured that the density of the bulk reservoir didn’t drop more than 1% of the initial density (calculated at 1 atm) to get accurate values for density inside the CNT. Once a constant density is obtained in the CNT, the reservoir was removed, and the fluid-filled CNT was used to carry out the flow simulation.

**Figure 8:**
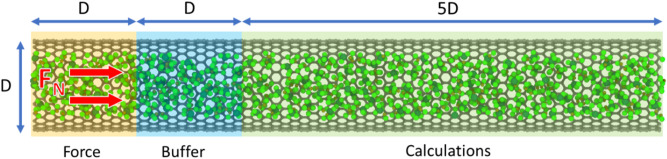
CNT filled with fluid and force ${\text{F}}_{{\text{N}}}$ applied on all the carbon atoms to simulate the flow. The blue dashed line represents the periodic boundary. Created using OVITO (ver:3.3.0).

For the flow simulation, the CNT filled with fluid (Fig. 8) was equilibrated for 1 ns at 300 K under NVT. The mean average displacement (MSD) of the molecules was calculated to measure the self-diffusion coefficient for the fluid. The data to calculate the mean squared displacement (MSD) was collected after every 1000 timestep (0.001 ns). The MSD was calculated using VMD, and the self-diffusion coefficient was calculated as per Eq. (6) for the three-dimensional (3D) system.

There are many ways to simulate pressure-driven flow across the flow channel. Jin and Firoozabadi^45,46^ used a dual control volume-grand canonical molecular dynamics (DCV-GCMD) method to simulate pressure difference across the pore. This method becomes computationally demanding as the size of the system increases. Another standard procedure is to apply an external force on all the atoms in the channel to create a pressure gradient^39,47–51^. However, this method affects the dynamics of the molecules and the resulting interference with the fluid wall interactions may lead to inaccurate results. In this study, we chose to use an approach that does not interfere with the fluid wall interactions and requires relatively low computational resources^52^, which is a modified method used by Wu and Firoozabadi^53^. The CNT length was divided into three regions, force, buffer, and calculation (see Fig. 8). The length of force and buffer region is equal to the tube’s diameter, and the length of the calculation region is five times the diameter. Then a constant force ($$F_{N}$$) was applied to all the carbon atoms present in the force region to simulate a pressure drop of 1 atm/Angstrom ($$\Delta P$$ across the tube length$$)$$ inside the CNT^39,47–52^. Since applying an external force interrupts the natural dynamics of molecules, applying an external force on a small region along with the buffer region allows the dynamics to be restored so that the calculations are performed on the molecules without the influence of the external force. The force, $$F_{N}$$ was equally distributed over the backbone (Carbon atoms for the hydrocarbon) of the fluid molecule. A periodic boundary was set in the flow direction (dashed blue line), allowing the CNT to be infinitely long (to allow fully developed flow). The applied force was calculated as per Eq. (4) for the given pressure drop per unit length, where “*n*_*T*_” represents the total number of fluid molecules in the CNT, “*V*” represents the volume of the CNT and “*L*” is the total length.

4 $$F_{N} = \frac{\Delta P}{L}\frac{V}{{n_{T} }}.$$

The simulation was allowed to run long enough to develop a fully developed flow inside the CNT—that is, when the average velocity in the direction of flow became constant. Once the flow is fully developed, the average velocities of all the molecules were averaged over time ($$v_{avg} )$$ in the calculation region. The average velocity of all the molecules was calculated over 500,000 timesteps (0.5 ns) until a constant value was obtained. In the case of smaller diameter CNT (1 nm and 2 nm), as the number of molecules is fewer, there is a lot of variation (noise) in the results, mainly while calculating density and velocity. Hence, a tolerance level of 4–5% was set for the smaller CNT and 1–2% (maximum deviations from the average) for larger CNT while calculating steady flow.

The average velocity was used to calculate the equivalent flowrate ($Q_{eq\_MD}$) values for all the cases by using Eq. (5), where “*μ*” represents the viscosity of the fluid and “*R*” is the radius of the tube. The equivalent flow is defined as the product of the viscosity of the fluid and its flow rate. This is done to make the results for different fluids with different viscosities comparable. For the Haggen Poisuelle (HP) flow, the equivalent $(Q_{eq\_HP}$) flow can be written by rearranging the Haggen Poisuelle equation, as shown in Eq. (6), where, “*R*” is the radius of the tube, “*Q*” is the HP flowrate, “$\Delta P/L$” is the pressure gradient across the tube.

5 $$Q_{eq\_MD} = \pi R^{2} \mu v_{avg} ,$$

6 $$Q_{eq\_HP} = Q \cdot \mu = \frac{{\Delta P\pi R^{4} }}{8L}.$$

The enhancement (E) in flowrate of fluid in CNT compared to the Hagen Poiseuille flow is defined as Eq. (7) in the existing literature.

7 $$E = \frac{{Q_{eq\_MD} }}{{Q_{eq\_HP} }}.$$

A comparative study between DCV-GCMD and section driven flow (SDF) to simulate a pressure driven flow in a confined channel is shown in the Supplementary Document.

## Supplementary Information

Supplementary Information.

## Nomenclatures

$A_{r}$: Cross-sectional area of the pore (Å^2^)
$A_{T}$: Cross-sectional area of the tube (Å^2^)
$d$: Number of dimensions
$D$: Self-diffusion coefficient (m^2^/s)
$$E$$: Enhancement factor
$F_{N}$: Force applied on each atom (Kcal/mole–Å)
$$k$$: Permeability (Å^2^)
$L_{c}$: Length of the pore (Å)
$L_{T}$: Length of the tube (CNT) (Å)
$M$: Molecular weight (g/Mol)
*n*: Number of molecules
$n_{T}$: Number of molecules inside the tube
$$N_{A}$$: Avogadro’s number
$Q$: Flow rate (pL/s)
$q_{darcy}$: Flow rate calculated as per the Darcy’s law (pL/s)
$Q_{eq\_HP}$: Equivalent flow rate from Haggen poiseulle (pL/s) (Pa s)
$Q_{eq\_MD}$: Equivalent flow rate from MD simulations (pL/s) (Pa s)
$$t_{o} - t$$: Time (s)
$V$: Volume of the simulation box (Å^3^)
$V\left( r \right)$: Interatomic potential at any distance “r” (kcal/mol)
$\Delta P$: Pressure drop (atm)
$\epsilon P$: Depth of Lennard–Jones (LJ) potential well (kcal/mol)
$$\mu$$: Viscosity (atm s)
$\rho$: Bulk density of fluid (g/Å^3^)
$\rho_{T}$: Density of the fluid inside the tube (g/Å^3^)
$\sigma$: Distance at which the inter-particle potential approaches zero (Å
