# Supplementary material for: Flow of long chain hydrocarbons through carbon nanotubes (CNTs)
Source: Sci Rep. 2021 May 26;11:11015. doi: 10.1038/s41598-021-90213-7 (PMC8155036; doi:10.1038/s41598-021-90213-7)
Supplement: Supplementary file 1 — Supplementary Information. [file 41598_2021_90213_MOESM1_ESM.docx]

**Supplementary information for**

**Flow of Long Chain Hydrocarbons Through Carbon Nanotubes (CNTs)**

Pranay Asai, Raul Velasco, Palash Panja, and Milind Deo

**Molecular Dynamics Simulation of Nano-Pores**

To study the effect of confinement on long chain hydrocarbons, a series of NEMD (Non-equilibrium Molecular Dynamics) simulations were performed with different pore diameters and different fluid types. The simulations were carried out using LAMMPS (Plimpton 1995). As per the theory, the choice of fluid and the material of the pore, should not affect the permeability.

In this study, the intermolecular interactions for the molecules are modeled using 6-12 Lennard–Jones (LJ) (Eq1). The $"\boldsymbol{\varepsilon"}$ represents the depth of the potential well and has the units of energy and $"\boldsymbol{\sigma"}$ represents the distance at which the inter-particle potential approaches zero and has the units of distance. The $"\boldsymbol{r"}$ terms are the distance between two particles. The LJ potential $\boldsymbol{"}\left( \boldsymbol{\sigma/r} \right)^{\boldsymbol{12}}\boldsymbol{"}$ term describes electron repulsion over the short-ranges and the $\boldsymbol{"}\left( \boldsymbol{\sigma/r} \right)^{\boldsymbol{6}}\boldsymbol{"}$ term describes the long-range attraction (van der Waals force) between atoms. The cutoff distance of 12Å was used for all the simulations. It has been shown that using a cutoff distance of more than $\boldsymbol{2.5}\boldsymbol{\sigma}$ has a negligible effect on simulation results [1].

|  | $V(r)= 4\epsilon\left[ \left( \frac{\sigma}{r} \right)^{12}-\left( \frac{\sigma}{r} \right)^{6} \right]$ | (1) |
| --- | --- | --- |

For the interactions between the same atom type, the values for $"\boldsymbol{\varepsilon"}$ and $"\boldsymbol{\sigma"}$ are chosen from the respective force fields. For the interaction between two different atoms (intermolecular interaction), the parameters are calculated using Lorentz-Berthelot combining rules (Eq 2 and Eq 3) [2]. Where $"i"$ and $"j"$represents the atom types.

| $\sigma_{ij}=\frac{\sigma_{ii}+\sigma_{jj}}{2}$ | (2) |
| --- | --- |
| $\epsilon_{ij}=\sqrt{\epsilon_{ii}\epsilon_{jj}}$ | (3) |

To perform the simulations, unlike the conventional method of using two-fluid boxes attached to the two ends of the CNT [3], another simple approach was designed to simulate the flow inside the CNT. A pore model was created (assuming a cylindrical pore) using a simple zig-zag carbon nanotube (CNT) with its length as approximately five times its diameter (to avoid end effects while filling the tube). This allows simulating the system with a significantly smaller number of fluid molecules, thus reducing the computational overhead. The CNT potentials were simulated using Optimized Potential for Liquid Simulation- All Atom (OPLS-AA) force field [4] and the CNT was considered rigid. This approximation can be justified by the high vibrational frequencies of a carbon atom as compared to the atoms of the fluid passing through it [5-7].

In this study, four CNTs of the different radius (Table 1) along with four different fluid, Water (H_2_O), Hexane (C_6_H_14_), Heptane (C_7_H_16_), and Decane (C_10_H_22_) are included. The water molecules were modeled using the extended simple point charge model (SPC/E) (Fig 1a) model [8]. For simplicity, the water molecule structure was held rigid using the SHAKE algorithm [9, 10] throughout the simulation. The Hexane, Heptane, and Decane (Fig 1b, 1c, & 1d) molecules were created using the OPLS-AA force field using moltemplate[11].

Table 1: The physical properties of CNT

| Sr. No. | Diameter (nm) | Length (nm) |
| --- | --- | --- |
| 1 | 1.01278 | 7.08946 |
| 2 | 2.03302 | 14.23114 |
| 3 | 5.00946 | 35.06622 |
| 4 | 8.02044 | 56.14308 |

**Comparison between DCV-GCMD and Section driven flow (SDF) simulation:**

In this study pressure driven flow inside the CNT is simulated by applying acceleration in a smaller region of the CNT and then measuring the flow properties in a different region. This section compares this method with an existing DCV-GCMD method used by Jin & Firoozabadi [12].

To perform the comparison, section driven flow models for methane flow in graphene slit (three layered), was created by following the directions from Jin & Firoozabadi (including the forcefield data). To goal was to compare the density and velocity profile for the two methods at high pressure flow. Three different model with the slit size of 1nm, 4nm and 10nm were created. The length to slit width ratio was maintained at 7:1. The slits were prefilled with methane by using the number density data obtained by Jin & Firoozabadi. The pressure gradients were also calculated from their study and the corresponding acceleration was applied to out to our models. The velocity was sampled every 20fs and the average velocity over 1ns was plotted after achieving a steady state.


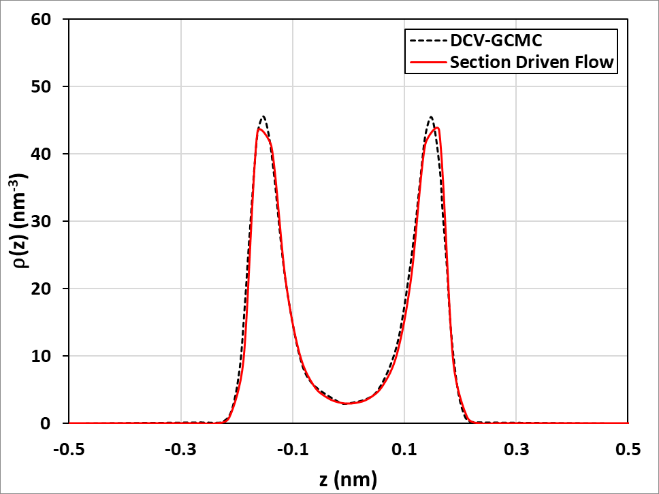

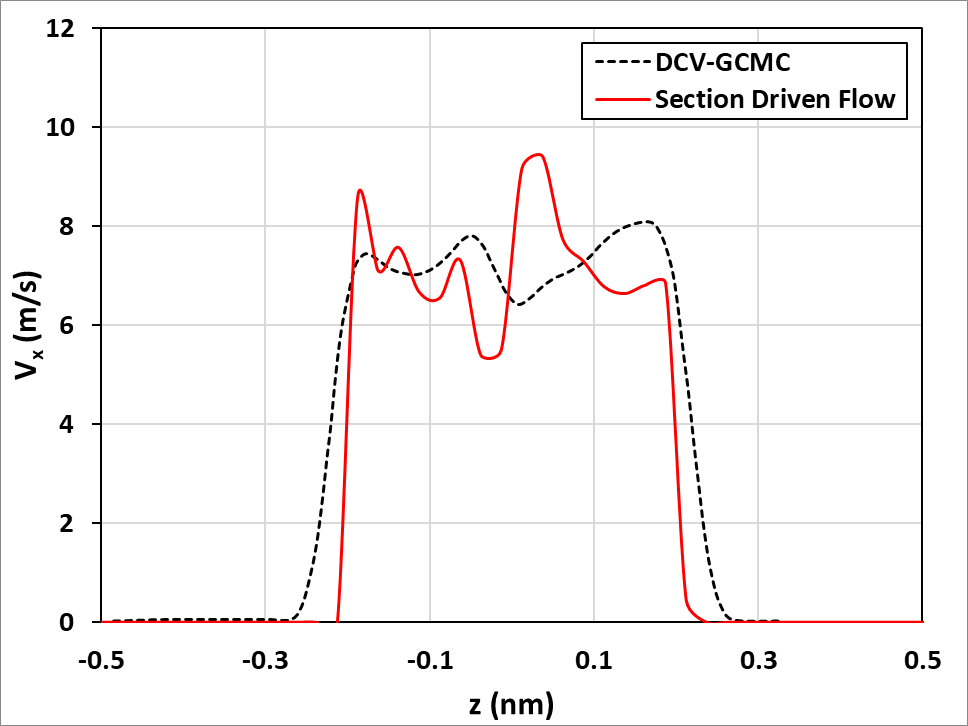


(a) (b)

*Figure 1: a) Comparison between density profile of methane molecules for DCV-GCMD and Section driven flow. b) Comparison between velocity profile (x-z) of methane molecules for DCV-GCMD and Section driven flow. SDF: Length=7nm, slit width =1nm, ΔP/L=0.044atm/A.*


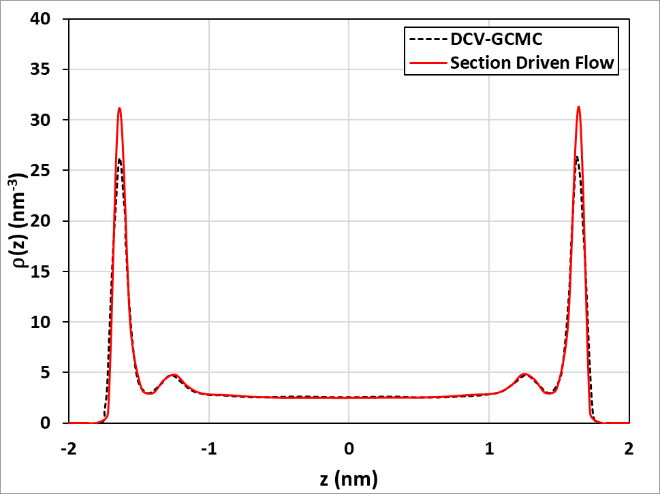

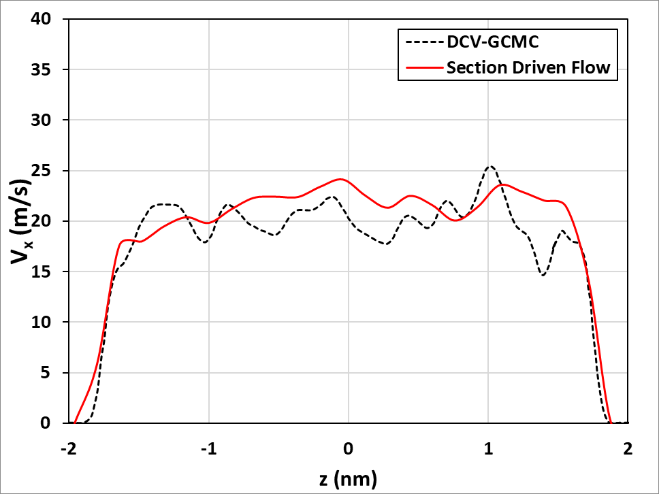


(a) (b)

*Figure 2: a) Comparison between density profile of methane molecules for DCV-GCMD and Section driven flow. b) Comparison between velocity profile (x-z) of methane molecules for DCV-GCMD and Section driven flow. SDF: Length=28nm, slit width =4nm, ΔP/L=0.022atm/A.*


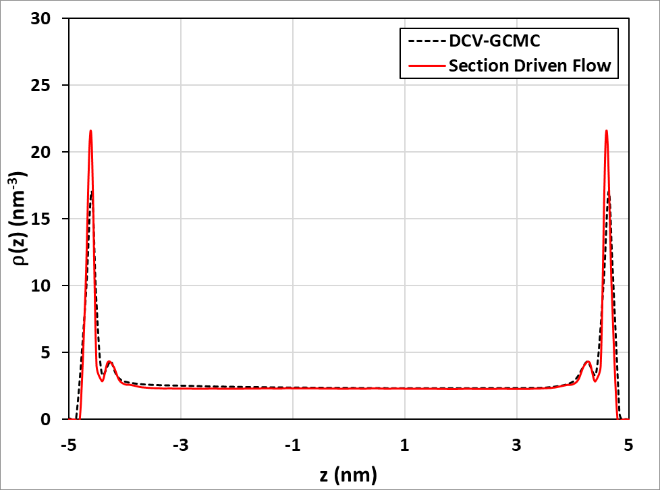

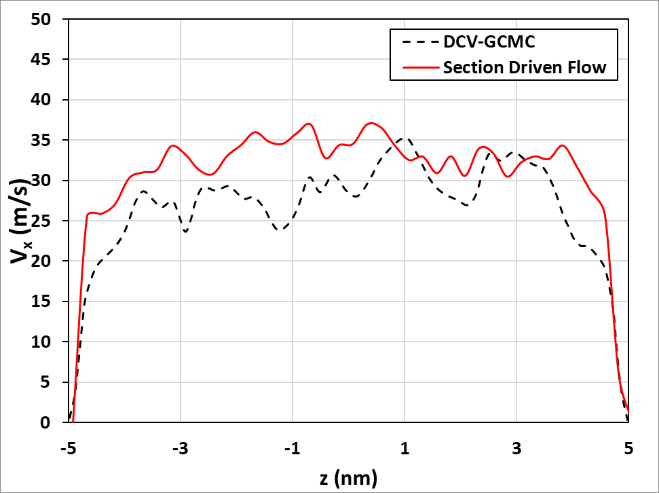


*Figure 3: a) Comparison between density profile of methane molecules for DCV-GCMD and Section driven flow. b) Comparison between velocity profile (x-z) of methane molecules for DCV-GCMD and Section driven flow. SDF: Length=70nm, slit width =10nm, ΔP/L=0.022atm/A.*

As seen from figures 1,2 and 3, the results for both the methods are almost identical, thus showing that section driven flow method can be successfully used to simulate flow in nanotubes/channels. The main advantage of using SDF model is that it require less number of molecules and hence allows to simulate larger pore size with less computational requirement.

**References**

1. Huang, C., et al., *Effect of cut-off distance used in molecular dynamics simulations on fluid properties.* Molecular Simulation, 2010. **36**(11): p. 856-864.

2. Nakanishi, K. and K. Toukubo, *Molecular dynamics studies of Lennard‐Jones liquid mixtures. V. Local composition in several kinds of equimolar mixtures with different combining rule.* The Journal of Chemical Physics, 1979. **70**(12): p. 5848-5850.

3. Huang, C., et al., *Molecular dynamics simulation of a pressure-driven liquid transport process in a cylindrical nanopore using two self-adjusting plates.* J Chem Phys, 2006. **124**(23): p. 234701.

4. Jorgensen, W.L. and J. Tirado-Rives, *The OPLS [optimized potentials for liquid simulations] potential functions for proteins, energy minimizations for crystals of cyclic peptides and crambin.* Journal of the American Chemical Society, 1988. **110**(6): p. 1657-1666.

5. Hanasaki, I. and A. Nakatani, *Flow structure of water in carbon nanotubes: poiseuille type or plug-like?* J Chem Phys, 2006. **124**(14): p. 144708.

6. Werder, T., et al., *Molecular Dynamics Simulation of Contact Angles of Water Droplets in Carbon Nanotubes.* Nano Letters, 2001. **1**(12): p. 697-702.

7. Werder, T., et al., *On the Water−Carbon Interaction for Use in Molecular Dynamics Simulations of Graphite and Carbon Nanotubes.* The Journal of Physical Chemistry B, 2003. **107**(6): p. 1345-1352.

8. Berendsen, H.J.C., J.R. Grigera, and T.P. Straatsma, *The missing term in effective pair potentials.* The Journal of Physical Chemistry, 1987. **91**(24): p. 6269-6271.

9. Ryckaert, J.-P., G. Ciccotti, and H.J.C. Berendsen, *Numerical integration of the cartesian equations of motion of a system with constraints: molecular dynamics of n-alkanes.* Journal of Computational Physics, 1977. **23**(3): p. 327-341.

10. Chatterjee, S., et al., *A computational investigation of thermodynamics, structure, dynamics and solvation behavior in modified water models.* J Chem Phys, 2008. **128**(12): p. 124511.

11. Jewett, A. [*http://www.moltemplate.org*](http://www.moltemplate.org) [cited 2019 1st November].

12. Jin, Z. and A. Firoozabadi, *Phase behavior and flow in shale nanopores from molecular simulations.* Fluid Phase Equilibria, 2016. **430**: p. 156-168.
